# Kaposiform Hemangioendothelioma: clinicopathological characteristics of 8 cases of a rare vascular tumor and review of literature

**DOI:** 10.1186/s13000-021-01080-9

**Published:** 2021-03-15

**Authors:** Qurratulain Chundriger, Muhammad Usman Tariq, Jamshid Abdul-Ghafar, Arsalan Ahmed, Nasir Ud Din

**Affiliations:** 1 Section of Histopathology, Department of Pathology and Laboratory Medicine, Aga Khan University Hospital, Karachi, Pakistan; 2 Department of Pathology and Clinical Laboratory, French Medical Institute for Mothers and Children (FMIC), Kabul, Afghanistan

**Keywords:** Kaposiform hemangioendothelioma, Kaposi sarcoma, Hemangioma, HHV8

## Abstract

**Background:**

Kaposiform Hemangioendothelioma (KHE) is a rare vascular tumor of intermediate malignant potential which shows locally aggressive growth but only rarely metastasizes. It is mostly considered to be a tumor of pediatric population but its occurrence in the adults is not uncommon as once considered. Histologically, KHE can mimic other soft tissue neoplasms of different behaviors (e.g. Kaposi Sarcoma, hemangioma) and establishing the correct diagnosis is important for appropriate treatment. Herein, we describe the clinicopathological features of 8 cases of KHE which will be helpful in making their diagnosis.

**Methods:**

We reviewed pathology reports, microscopy glass slides and obtained follow up information about 8 cases of KHE which were diagnosed at our institution from January 2008 till June 2020. Immunohistochemical stain for HHV8 was also performed.

**Results:**

Age ranged from 7 months to 25 years. Seven patients were less than 20 years of age and one patient was 25 years old. Equal gender distribution was observed. Extremities were the most common sites of involvement, followed by head and neck, pancreas and ischiorectal region. 2 cases were resection specimen and all others were incisional biopsies. The largest tumor size was 5.5 cm in one of the resections. The incisional/fragmented tissues were all less than 5 cm in aggregate. Most cases showed predominance of nodular growth and a minor component of spindle cell population along with lymphangiomatosis like vascular channels, with evidence of microthrombi in 2 cases. Few multinucleated giant cells were observed in 2 cases. None of the cases exhibited significant nuclear atypia or mitotic activity. One of the cases arising in dermis showed underlying bone involvement. HHV8 was negative in 7/7 cases.

**Conclusions:**

KHE can also involve adult population and it should always be considered in the differential diagnoses of a vascular lesion. Presence of multinucleated giant cells is a rare finding. Knowledge about histological features and potential mimics is helpful in avoiding misdiagnosis.

## Introduction

According to the recent World Health Organization (WHO) classification of soft tissue tumors, vascular tumors are categorized as benign, intermediate malignant potential and outright malignant based on risk of metastasis and local recurrence. The intermediate malignant potential category is further categorized into tumors which are locally aggressive with a high rate of local recurrence and those which rarely metastasize to distant organs. Kaposiform Hemangioendothelioma (KHE) belongs to the former subcategory of vascular tumors with intermediate malignant potential. KHE shows histological features overlapping with benign hemangioma and Kaposi Sarcoma (KS) [1]. It is traditionally considered to be a tumor of infants and children, but many adult cases have also been reported in the literature [2]. Distal extremities are the classic sites but many unusual sites including head and neck region and viscera have also been reported [3]. Once considered to be rare, the literature search does yield some large series, the largest reporting 100 cases from a single institution [4]. Microscopically, it shows combination of nodular and spindle cell growth patterns in varying proportions, which may be surrounded by ectatic lymphatic channels resembling lymphangiomatosis in some examples [2]. The nodules are glomeruloid aggregates of vascular channels, with variable slit-like lumina and surrounding pericytic layer. The neoplastic cells have pale eosinophilic cytoplasm and uniform appearing nuclei with folded contours and none to minimal atypia [1]. Based on glomeruloid structures, KHE needs to be differentiated from infantile hemangioma and tufted angioma, both of which are benign tumors of pediatric population. The spindle cell component resembles and needs to be differentiated from KS which is a malignant tumor. Human Herpes Virus-8 (HHV-8) immunohistochemical (IHC) stain helps in distinguishing KS from KHE as it is positive in KS while negative in KHE [5].

In this study, we have described the clinical and histopathological characteristics of 8 cases of KHE which would be helpful in making their correct diagnosis. We also attempted to obtain follow up information in order to understand the behavior and prognosis of this tumor.

## Materials and Methods

We searched the surgical pathology database of our institution through Integrated Laboratory Management System (ILMS) software for cases diagnosed as “KHE” from January 2008 till June 2020. Approval by the Institutional Ethical Review Committee (ERC # 2020-5031-13950) was sought.

Patient demographics including age, gender, tumor site and clinical presentation were recorded from surgical pathology reports. Hematoxylin & eosin stained (H&E) and IHC stained slides of these cases were retrieved from the archives and reviewed by 2 pathologists to observe histological features including the respective percentages of nodular and spindle components, nuclear atypia, presence of multinucleated giant cells, extent of tumor invasion, mitotic activity, presence of lymphatic channels, margin status and other unusual morphological features. Formalin fixed paraffin embedded tissue blocks were stained with HHV-8 antibody (Clone 13B10 by Cell Marque) according to the manufacturer’s protocol by DAKO envision system. All cases were also stained with CD31, CD34, ASMA and ERG antibodies. At least 5 % of the tumor cells showing intermediate to strong nuclear positivity was taken as the cutoff for HHV-8 positivity [6].

Information regarding subsequent treatment and follow up was obtained from patients via telephonic conversation.

## Results

Clinical features: A total of 8 cases were diagnosed during the study period. Table 1 summarizes the features and follow up information of these 8 cases. 7 of the 8 patients were younger than 20 years of age and only a single was an adult female. Male to female ratio was 1:1. Five cases involved distal extremities. One of these cases (case 2) was limited to the dermis while the rest involved deeper soft tissue. Another case (case 6) showed infiltration into underlying bone.

**Table 1:** Summary of clinical features and follow up information of KH patients (*n* = 8)

Case #	Patient’s age	Gender	Tumor Site	Presenting feature	Follow up duration	Follow-up status
1	7 months	Male	Calf	Not mentioned	144 months	Alive and disease free
2	6 years	Female	Skin over shin	Ulcer	120 months	Alive and disease free
3	10 years	Female	Nose	Not mentioned	120 months	Alive and disease free
4	2 years	Female	Metacarpals	Not mentioned	96 months	Alive and disease free
5	8 months	Male	Synovium knee	Not mentioned	84 months	Alive and disease free
6	4 years	Male	Thigh	Not mentioned	40 months	Lost to follow up
7	18 years	Male	Pancreas	Common bile duct obstruction	24 months	Alive and disease free
8	25 years	Female	Ischiorectal area	Indurated skin	12 months	Alive and disease free

Out of the remaining 3 cases, one arose in the head of pancreas of an 18-year-old male with classic clinical signs and symptoms of common bile duct obstruction i.e. pruritus, jaundice and weight loss. Computed Tomography (CT) scan showed intra and extra-hepatic biliary dilatation along with moderate dilatation of the pancreatic duct. There was a focus of ill-defined enhancement in the peri-ampullary region which indented the common bile and pancreatic ducts resulting in their dilatation. Patient underwent Whipple’s procedure for tumor excision. Second case involved the nose of a 10-year-old female and third case involved the ischiorectal region of the only adult patient of our cohort.

2 cases were resection specimen and all others were incisional biopsies. The largest tumor size was 5.5 cm in one of the resections. The incisional/fragmented tissues were all less than 5 cm in aggregate (Table 2). Tumor was not associated with Kassabech-Meritt Syndrome/Phenomenon (KMP) in any of the cases. Data regarding KMP was obtained from the laboratory reports such as complete blood count, prothrombin time and international normalized ratio (INR), available on ILMS software. Patients were also inquired through telephonic conversation about their presenting complaints or bleeding crisis.

**Table 2:** Summary of histological features of KH cases. (*n* = 8)

Case #	Tumor size (cm.)	Percentage of Vascular Nodules	Percentage of Spindle cells	Lymphangioma-like vessels	Mitoses /10 HPFs	Cytoplasmic vacuoles	Microthrombi	Extent/Plane of invasion (Skin, subcutaneous tissue, bone)	Margins	Additional features
1^#^	5 × 2	5 %	95 %	None	6	Absent	Absent	Muscle	Involved	Sieve-like architecture of nodules
2	1 × 0.5^b^	> 95 %	< 5 %	None	5	Absent	Absent	Dermis	Involved	Lymphoid follicles, mixed inflammation
3	1.5 × 1^a^	80 %	20 %	None	7	Present	Absent	Dermis & subcutis	Cannot be assessed^a^	Hyaline globules
4	3 × 1.5^a^	> 95 %	None	< 5 %	6	Present	Absent	Soft tissue	Cannot be assessed^a^	Hemorrhage & Perineural invasion
5	2 × 0.8	80 %	15 %	5 %	4	Absent	Absent	Synovium	Involved	Background fibrosis
6	5.5 × 3.5	70 %	None	30 %	6	Present	Absent	Bone	Involved	Perineural invasion
7	1.5 cm.	80 %	15 %	5 %	6	Present	Present	Pancreas	Tumor free	None
8	2.5 × 1^b^	90 %	10 %	None	2	Present	Present	Dermis	Cannot be assessed^b^	None

^a^Tumor was received in multiple fragments

^b^Incisional biopsy specimen was received

### Pathological features

**Fig. 1:**
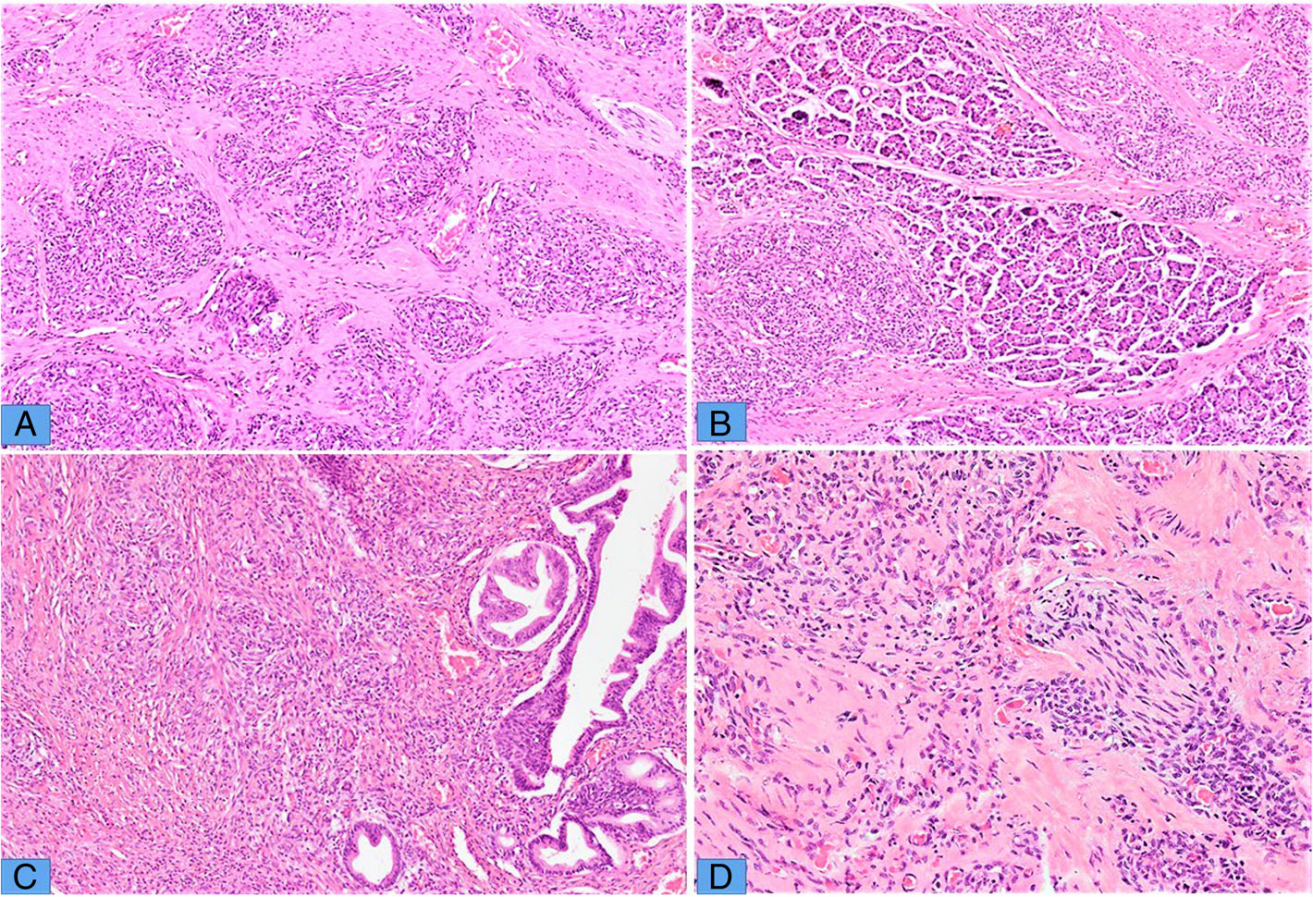
Pancreatic KHE. **a** H&E stained sections of tumor showing predominantly glomeruloid arrangement in this field. These glomeruloid nodules are composed of closely packed vascular channels lines by single layer of bland endothelial cells. **b** This area shows predominantly spindle cell growth, infiltrating into the pancreatic acini. These areas resemble KS. **c** Other areas of spindle cell growth surrounding lining of a pancreatic duct, where the spindled endothelial cells grow in sheets with slit like lumina containing variable number of erythrocytes. **d** Focus of perineurial invasion

**Fig. 2:**
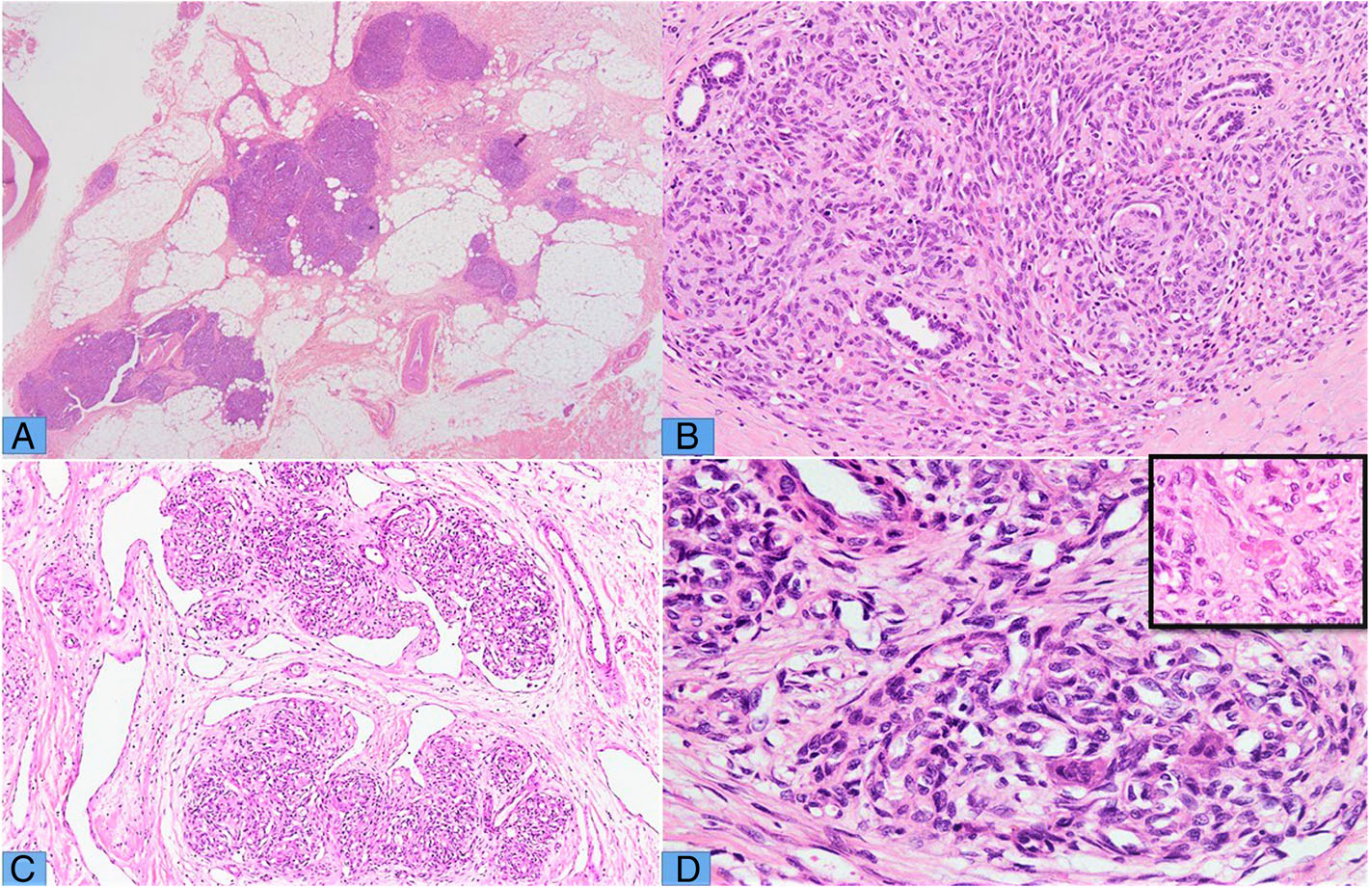
**a** H&E stained sections of cutaneous KHE, showing glomeruloid nodules of tumor infiltrating into subcutaneous adipose tissue. **b** In the dermis, the tumor nodules infiltrate through adnexal structures with entrapment of residual bi-layered eccrine glandular acini. **c** Tumor nodules with surrounding lymphangioma-like vascular spaces. **d** Tumor nodules showing few scattered multinucleated giant cells, which is a rare finding in KHE. D Inset) Microthrombi seen within capillary lumen

In all cases, tumors were predominantly composed of vascular nodules along with minor component of spindle cell areas and lymphatic vessels as described in Table 2. These vascular nodules comprised of endothelial cells with variable slit-like lumina and cytoplasmic vacuoles (Fig. 1 a-c). Mitotic figures ranged from 2 to 7 per 10 high power fields (HPFs). The nuclei appeared to be uniform with folded contours, fine chromatin and occasional small nucleoli. Perineurial invasion was seen in 2 cases (Fig. 1d). Tumors involving extremities showed infiltrating nodules of tumor into dermis and subcutaneous fat (Fig. 2 a) along with adnexal involvement (Fig. 2b). Significant nuclear atypia and necrosis were not seen in any of the cases. One of the cases showed prominent lymphatic vessels surrounding the vascular nodules (Fig. 2 c). Few intratumoral multinucleated giant cells were seen in 2 of our cases (Fig. 2d). This finding has not been described previously with KHE. Microthrombi were seen in 2 of our cases (Fig. 2d inset). The histological features of individual cases are summarized in Table 2.

**Fig. 3:**
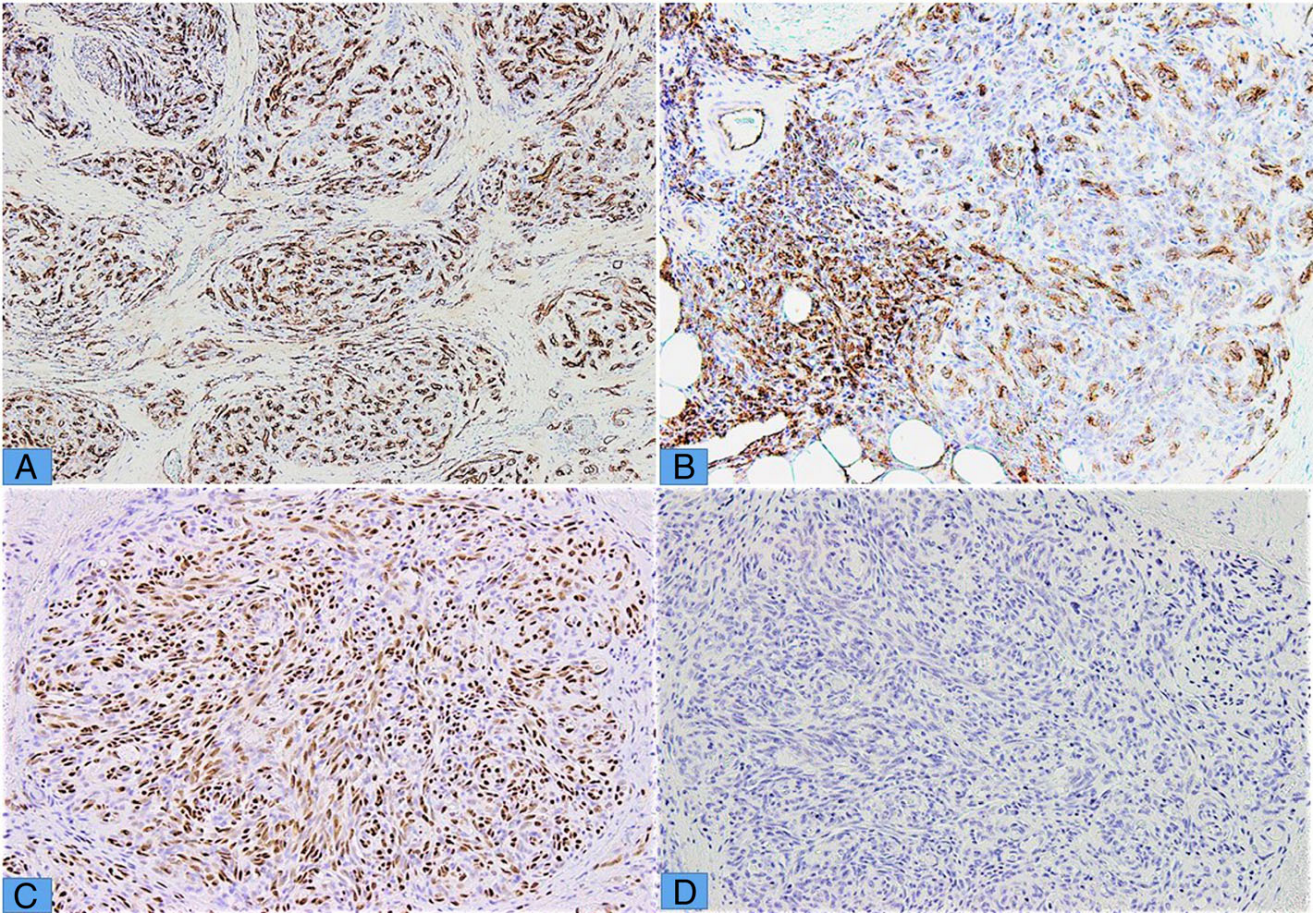
**a** & **b** CD34 and CD31 highlighting endothelial cells. **c** Nuclear staining of ERG in endothelial cells **d** Negative staining of HHV8

CD34, CD31 and ERG showed staining in endothelial cells (Fig. 3 a-c) and ASMA which showed staining in pericytic cells surrounding the vascular lumina. HHV-8 was negative in 7 cases in which it was performed (Fig. 3d). Immunohistochemical markers for lymphatic channels, like D2-40, LYVE-1 and VEGFR3 were not performed, as none of these markers are available in our laboratory.

### Follow‐up

7 out of 8 patients in our series were alive and disease free at the time of obtaining last follow up. None of them developed recurrence or metastasis. One of the patients was lost to follow up after 40 months (Table 1).

## Discussion

KHE was first described by Zukerberg et al. in 1993 as a rare vascular tumor of infancy and childhood, arising most commonly in deep soft tissue of extremities [2]. They delineated the clinical and pathological features of this distinctive tumor entity in a series of 9 cases [2]. Before that study was published, KHE was reported as individual case reports with vague names like “Kaposi-like Infantile Hemangioendothelioma” and “Epithelioid and spindle cell Hemangioendothelioma” [7, 8]. In the series reported by Zukerberg et al., 7 out of 9 patients were less than 10 years of age and none was older than 20 years [2]. Subsequent series and case reports described wide age range starting from antenatal period up to 46 years [9]. In our series, majority of the patients were children and the age ranged from 7 months to 25 years (Table 1).

Upper and lower extremities are the most common sites [2, 4]. This has been followed by mediastinum, retroperitoneum and head and neck region [4, 10–12]. In 2009 Fernandez Y et al. published a review of 165 KHE cases reported since the first series published in 1993 [3]. Patients ranged from newborns to 64 years of age, the majority of which were children under 12 years old, concordant with the earlier descriptions by Zuckerberg et al. [2]. Around 60 cases reported in that series involved extremities, the rest involved a multitude of sites [3]. Several studies have described KHE at varied sites or origin with unusual clinical presentations. Pancreas is one of the rare sites for KHE and so far, it has only been reported in 4 cases, all of these patients being less than 2 years of age [13–16]. One of the cases in our series also involved pancreas. The patient was 18-year-old male who presented with pruritus, jaundice and weigh loss. On CT scan, tumor appeared as an ill-defined enhancement in the peri-ampullary area which was causing dilatation of intra and extra-hepatic biliary ducts and pancreatic ducts. He underwent Whipple’s procedure and was alive and tumor free after 24 months follow up duration. Other rare tumor sites with unusual clinical presentation have also been described in individual case reports such as a tumor arising in antecubital fossa at the site of previous trauma and a fatal tumor inside the cranial cavity centered on the tentorium cerebelli [17, 18].

In the dermal and/or subcutaneous locations, KHE clinically appears as a reddish to violet plaque or nodule, that can grow deep down into the soft tissue or even bone, as seen in one of our cases which showed bone infiltration (case 6). In deeper locations, the clinical symptoms depend on the site of tumor involvement. If tumor involves deep fascial planes, it infiltrates as nodules with surrounding desmoplastic response and it may present with mass effect. If the tumor involves retroperitoneum or visceral organs, the presentation may be with thrombocytopenic coagulopathy or KMP, which is commonly associated with this tumor. In the latter scenario, the tumor may come to attention after workup of coagulopathy [13].

For our patients, data regarding associated KMP was collected over telephonic conversation by asking leading questions like if there was any history of bleeding or anti-coagulant therapy before or during hospital stay. The information obtained thus concluded that none of the cases in our cohort had associated KMP.

Histologically, KHE predominantly comprises of glomeruloid proliferation of vascular channels lined by endothelial cells which are positive for vascular markers such as CD31 and CD34 [1]. These structures are the site of sequestration and destruction of red blood cells and platelets [5]. Smooth muscle actin positive pericytic cells can also be focally seen surrounding these endothelial cells. Adjacent to these areas, there are ill-defined zones of lymphatic channels which are found to be positive for markers of lymphatic differentiation such as D2-40, LYVE-1 and VEGFR3. These foci may also exhibit spindle cell proliferation which can mimic KS with formation of slit-like spaces. Distinction from KS is done by the presence of glomeruloid proliferations and absence of HHV-8 by either IHC or molecular methods to detect DNA of the Human Herpes virus 8 [5]. Intracytoplasmic vacuoles may be seen in the neoplastic cells, which is a common finding in vascular neoplasms [19].

We observed intratumoral multinucleated giant cells in 2 of our cases. To the best of our knowledge, this finding has not so far been reported in the literature within KHE. Though studies have described presence of multinucleated giant cells within other vascular neoplasms [20]. Studies conducted on cases of vasculitis have demonstrated the multinucleated giant cells and other CD68 positive macrophages as sources of vascular endothelial growth factor (VEGF) and interferon gamma, both of which are mediators of angiogenesis [21]. The presence of these cells thus theoretically may be one of the underlying mechanisms of vascular proliferation and tumorigenesis in vascular neoplasms.

Juvenile Hemangioma (JH) can show similar presentation in the pediatric population but it lacks the typical rounded nodules of KHE, lacks associated red cell sequestration and KMP. GLUT1 also helps in distinction as it is usually expressed in JH while it is negative in KHE. Tufted Angioma (TA) also presents in the same pediatric population in the skin as erythematous and violaceous plaques. Histologically, it also shows cannon ball-like clusters of capillary sized blood vessels growing within the dermis [22]. Some authors suggest these 2 entities represent two ends of the histological spectrum where TA is at the benign end while KHE is at the aggressive end [4]. On this theme, the recent WHO classification of tumors of the soft tissue, the two entities have been combined finally after a long debate [1]. We also evidenced aggressive local growth in the form of underlying bone infiltration in one of our cases.

Almost all pediatric cases of KHE described in the literature had been associated with KMP, particularly those involving deeper soft tissues such as retroperitoneum and mediastinal cavities [11], though KMP is not exclusive to these tumors and can also be seen with some superficial tumors arising in the dermis and subcutis [4]. KMP is characterized by thrombocytopenic coagulopathy and was first described by Kassabach and Merritt in 1940 as purpura developing in patients with capillary hemangioma [23]. KHE rarely causes death due to mass effect or metastasis; however, the presence of KMP is associated with significant mortality in these patients [24]. Interestingly, symptoms and laboratory findings related to KMP were not observed in any case of our study and only 2 cases showed evidence of microthrombi. In 2005, Gruman and colleagues described 10 cases of KHE who presented without KMP. All presented as a skin covered soft tissue mass and the tumor size was less than 8 cm. They found microscopic evidence of platelet trapping and hemosiderin deposition. They postulated that the lack of clinical appearance of KMP can be related to small tumor size [25]. Similarly, in another study, Schmidt et al. reviewed 231 cases of KHE and found that the size of KHE cases without KMP was less than the cases with KMP (12 cm^2^ versus 49 cm^2^). They also noticed that the incidence of KMP was highest in the infants and it decreased with increasing age. The cases with KMP were located in the retroperitoneum and extra-regional sites [26]. Most of our cases were incisional biopsies and few were sent as multiple fragments of tumor tissue, the largest tumor size in our study was 5.5 cm in the greatest dimension, which is less than 8 cm as described by Gruman et al. [25]. With regards to patinets’ age at presentation, our cohort comprised of 7 of 8 cases being less than 20 years of age. The oldest was also young (25 years old). This finding though contrasts with the literature, we are of the opinion that the lack of KMP in our cases can be related to small tumor size. Further studies are needed to find an explanation for this unusual phenomenon.

Like other vascular tumors exhibiting intermediate malignant behavior, KHE shows aggressive local growth with infiltration into more than one tissue planes and it may also recur after incomplete excision. Spontaneous regression is not known to occur. Complete surgical removal with wide margins is the most common treatment modality. Combination of corticosteroids and vincristine is administered for unresectable tumors [27]. Some studies have also reported good response with interferon alpha and sirolimus while others didn’t experience any response to the latter drug [15, 28]. Control over consequences of KMP may require administration of blood products like fresh frozen plasma and platelets [29].

## Conclusions

KHE is an extremely rare vascular tumor which shows aggressive local growth and may be associated with KMP. Pediatric population is the most commonly affected; however, adults can also be affected. Apart from classic presentation in an extremity, rare sites can also be involved. It may not always be associated with KMP, particularly in older patients and those with smaller tumors. It should always be considered in the differential diagnoses of vascular neoplasms in all age groups. Thorough knowledge of histological features of KHE and its mimics is helpful in reaching accurate diagnoses and administering appropriate treatment and management.

## Data Availability

Data and materials of this work are available from the corresponding author on reasonable request.

## Abbreviations

KHE: Kaposiform Hemangioendothelioma
WHO: World Health Organization
KS: Kaposi sarcoma
HHV-8: Human Herpes Virus-8
IHC: Immunohistochemical
ILMS: Integrated Laboratory Management System
ERC: Ethical Review Committee
H&E: Hematoxylin & eosin
CT: Computed Tomography
KMP: Kassabech-Meritt Syndrome/Phenomenon
INR: International normalized ratio
HPF: High power field
VEGF: Vascular endothelial growth factor
JH: Juvenile hemangioma
TA: Tufted angioma
